# Dynamic Causal Modeling of the Cortical Responses to Wrist Perturbations

**DOI:** 10.3389/fnins.2017.00518

**Published:** 2017-09-13

**Authors:** Yuan Yang, Bekir Guliyev, Alfred C. Schouten

**Affiliations:** ^1^Neuromuscular Control Laboratory, Department of Biomechanical Engineering, Delft University of Technology Delft, Netherlands; ^2^Department of Physical Therapy and Human Movement Sciences, Feinberg School of Medicine, Northwestern University Chicago, IL, United States; ^3^Department of Biomechanical Engineering, MIRA Institute for Biomedical Technology and Technical Medicine, University of Twente Enschede, Netherlands

**Keywords:** sensory feedback, stretch response, dynamic causal modeling, sensorimotor network, EEG, effective connectivity

## Abstract

Mechanical perturbations applied to the wrist joint typically evoke a stereotypical sequence of cortical and muscle responses. The early cortical responses (<100 ms) are thought be involved in the “rapid” transcortical reaction to the perturbation while the late cortical responses (>100 ms) are related to the “slow” transcortical reaction. Although previous studies indicated that both responses involve the primary motor cortex, it remains unclear if both responses are engaged by the same effective connectivity in the cortical network. To answer this question, we investigated the effective connectivity cortical network after a “ramp-and-hold” mechanical perturbation, in both the early (<100 ms) and late (>100 ms) periods, using dynamic causal modeling. Ramp-and-hold perturbations were applied to the wrist joint while the subject maintained an isometric wrist flexion. Cortical activity was recorded using a 128-channel electroencephalogram (EEG). We investigated how the perturbation modulated the effective connectivity for the early and late periods. Bayesian model comparisons suggested that different effective connectivity networks are engaged in these two periods. For the early period, we found that only a few cortico-cortical connections were modulated, while more complicated connectivity was identified in the cortical network during the late period with multiple modulated cortico-cortical connections. The limited early cortical network likely allows for a rapid muscle response without involving high-level cognitive processes, while the complexity of the late network may facilitate coordinated responses.

## Introduction

Bodily movement is one of the main ways how humans interact with the physical world (Schwartz, 2016). Movement can be generated by voluntary and reflex driven actions. Muscle stretch during active motor task (e.g., maintain an isotonic wrist flexion) results in a sequence of cortical and muscle responses, involving the central nervous system and the periphery.

In the periphery, the immediate muscle responses to stretch are known as stretch reflexes. Many studies investigated muscle responses to stretch using electromyography (EMG) after ramp-and-hold mechanical perturbations. For lower arm muscles, they typically reported a short-latency stretch response (20–50 ms post-perturbation onset) followed by a long-latency stretch response (50–120 ms) and later voluntary reactions (>120 ms) (Scott, 2002; Pruszynski et al., 2011). The short-latency stretch response depends on the stretch velocity and involves a spinal network (Houk et al., 1981). The time delays in the afferent pathway from the periphery to the brain (20–30 ms) (MacKinnon et al., 2000) and the efferent pathway from the brain to the periphery (~20 ms) (Perenboom et al., 2015) would not allow for a transcortical pathway in the short-latency stretch response. Several experimental studies indicated cortical contributions to the long-latency stretch response. Recordings from cortico-motoneuronal cells in Macaque monkeys showed a cortical effect on the long-latency stretch response (Cheney and Fetz, 1984). Subthreshold transcranial magnetic stimulation (TMS) over the contralateral motor cortex can modulate the long-latency stretch response but not the short-latency stretch response (Perenboom et al., 2015). Recent studies indicate the long-latency stretch response is not as simple as a “reflex” and at least could partly involve a voluntary feedback control component (Pruszynski et al., 2011; Pruszynski and Scott, 2012). Thus, we avoid the term “reflex” and “voluntary” in this paper and use “rapid” and “slow” transcortical muscle reactions to roughly distinguish the cortical-involved muscle reactions before 120 ms (i.e., long-latency stretch response) and after 120 ms (i.e., “standard” voluntary reaction) post-perturbation. Similar terminology has been previously used in a review from Pruszynski and Scott (2012).

In the central nervous system, cortical responses to muscle stretch have been investigated by previous studies using the event-related potential (ERP) (Abbruzzese et al., 1985; Campfens et al., 2015). The latencies and topographies of the stretch-evoked ERP reflect the time courses of cortical activity and most active areas in response to the muscle stretch. Both early (<100 ms post-perturbation onset) and late (>100 ms) ERP components were reported around the contralateral motor cortex (Campfens et al., 2015). Considering the efferent motor conduction delay (~20 ms), the early cortical response is thought to related to the rapid transcortical muscle reaction to the perturbation (<~120 ms) while the late cortical response may be related to the slow transcortical muscle reaction (>~120 ms) (MacKinnon et al., 2000; Pruszynski and Scott, 2012). ERP results indicate that the primary motor cortex may contribute to both rapid and slow transcortical muscle reactions; however, exact cortical pathways are yet to investigate. The full cortical network for motor control is thought to involve multiple brain areas, including primary somatosensory cortex (S1), primary motor cortex (M1), premotor cortex (PM), supplementary motor area (SMA), and posterior parietal cortex (PPC) (Scott, 2004; Szameitat et al., 2012). These regions constitute the cortical sensorimotor network, which is a distributed and adaptable network that orchestrates the overall human motor behavior (Scott, 2004; Shibasaki, 2012).

In this study, we used dynamic causal modeling (DCM) to model the effective connectivity in the cortical network modulated by muscle stretch. Effective cortical connectivity refers to the strength of the causal influences between multiple cortical areas, which can be modulated by external perturbations (Friston, 2011). A few studies suggested that the rapid and slow transcortical muscle reactions are engaged by similar neural circuitries in the brain (Pruszynski et al., 2011; Pruszynski and Scott, 2012). However, we hypothesize that the early response engages effective cortical connectivity in a less complex network to accelerate the muscle response with a shorter delay, i.e., rapid transcortical muscle reactions; while the slow transcortical muscle reaction is governed by a more complex cortical network in the late cortical response.

To valid our hypothesis, we estimated effective connectivity among the cortical areas involved in sensorimotor control of the wrist in response to a perturbation. Previous studies considered only M1, SMA, and PM as “key motor regions” for upper limb movement (Grefkes et al., 2008; Chen et al., 2010). In line with review papers on feedback based motor control (Scott, 2002, 2004), we added S1 and PPC to our possible functional cortical network models, since these two areas are closely related to feedback-based motor control. S1 is the brain area receiving the peripheral somatosensory input, while the PPC is known as a sensory association area which is essential to integrate different sensory inputs. We investigated effective cortical connectivity in the early period within 100 ms post-perturbation onset in comparison to the late period between 100 and 350 ms post-perturbation onset to check if the rapid and slow transcortical muscle reactions involve similar cortical areas and signal propagation pathways. Considering the afferent sensory transmission time delay (~20 ms) (Abbruzzese et al., 1985; Campfens et al., 2015), we used 20–100 ms as the period to investigate the early cortical network.

## Materials and methods

### Subjects and ethical statement

Seven healthy right-handed volunteers (one female) aged 23–28 years old participated in the experiment. This study was carried out in accordance with the recommendations of Human Subject Research guidelines, the Human Research Ethics Committee of the Delft University of Technology with written informed consent from all subjects. All subjects gave written informed consent in accordance with the Declaration of Helsinki. All subjects signed informed consent before the experiment and received a small financial compensation for their participations. The protocol was approved by the Human Research Ethics Committee of the Delft University of Technology.

### Experimental protocol

Subjects sat next to a wrist manipulator (Wristalyzer, Moog Inc., the Netherlands), which is an actuated rotating device with a single degree of freedom to exert flexion and extension perturbations to the wrist joint. The lower arm of the subject was strapped in the armrest, while the subject was closely touching the handle of the wrist manipulator (fixed with velcro). Subjects were instructed to relax their fingers and only use the wrist to do the task. The axis of wrist manipulator rotation was aligned with the axis of wrist rotation. Wrist torque was measured by a force transducer within the handle of the wrist manipulator.

The protocol contained 30 trials. Each trial started with auditory cue “beep” and a fixation in the center of the screen with a random period of 1.5–2 s. After this random period, visual feedback was provided with an arrow in a circle. The angle of the arrow is proportional to the (low-pass filtered 1 Hz) torque applied by subjects. Subjects were instructed to push with a constant flexion torque (1.0 Nm) to the handle with their right wrist (keeping the arrow pointing upwards) using the visual feedback. Each trial contains 20 flexion and 20 extension ramp perturbations. Note that the visual feedback was low-pass filtered to avoid fast visual corrections to the perturbations. The wrist manipulator applied angular ramp perturbation to stretch the wrist muscles when the subject maintained the constant flexion torque (with std. <5%) for a random period of 1.5–2 s, and then stopped (and held) at the new position until the next perturbation. A ramp duration of 40 ms was used with the velocity 1.5 rad/s; giving a ramp amplitude of 0.06 rad. This duration is below the expected saturation level of long-latency EMG response and allows for both inhabitation and facilitation of the long-latency stretch response (Lee and Tatton, 1982; Meskers et al., 2009; Perenboom et al., 2015). During the ramp, subjects were instructed to maintain the same level of force. Since the subjects were required to maintain a flexion torque, only the data from the extension ramp perturbations (stretching the wrist flexors) were included for analysis.

Electroencephalogram (EEG) was recorded using a 128-channel cap (5/10 systems, WaveGuard cap, ANT Neuro, The Netherlands) with Al/AgCl electrodes. EMG signals were measured from the flexor and extensor carpi radialis muscles of the right forearm using bipolar derivations 2 cm inter-electrode distance. EEG signals were recorded at by a bio-signal amplifier (Refa System, TMSi, The Netherlands), which acquired data at a sampling frequency of 2,048 Hz. The amplifier contains an antialiasing low-pass filter with the cut-off frequency of 552 Hz.

### Data preprocessing

The continuous EEG signals were filtered by a 0.5–100 Hz zero-phase shift band-pass filter using EEGLAB (Delorme and Makeig, 2004) to remove possible high-frequency noise and slow trends in the data (e.g., blood pressure, heartbeat, breathing). A notch filter was used to reject the 50 Hz line power noise. Afterwards, EEG were segmented into 570 ms epochs with 220 ms pre-stimulus baseline plus 350 ms post-stimulus recording. The epochs contaminated by the artifacts (e.g., eye blinks/movements and EMG artifacts) were removed by visual inspection. In the data, we did not see visible artifacts due to the transient perturbations. On average 118 epochs were removed per participant, leaving 472 ± 53 epochs per participants for analysis. Then the ERPs were derived by grand averaging the remaining epochs using the period of 220–20 ms before stimulus onset as the baseline. These extracted ERPs corresponding to the neural activity in the cortical regions of interest are used to quantify effective connectivity between those regions via DCM.

### Dynamic causal modeling

DCM was applied to analyse the effective cortical connectivity. Although various methods are available for analyzing effective cortical connectivity, most of them focus on the linear connectivity, such as partial directed coherence (Kaminski and Blinowska, 1991; Porcaro et al., 2013) and directed transfer function (Babiloni et al., 2005). Previous studies have reported non-linear neuronal coupling in human stretch responses (Yang et al., 2016b) and voluntary motor control (Chen et al., 2010; Yang et al., 2016a) of lower arm muscles. Different from linear connectivity methods, DCM is a non-linear identification approach to reveal how external inputs cause changes in the coupling of neural populations in the effective connectivity network (Friston et al., 2003; Goulden et al., 2014).

We used the standard DCM for ERP (David et al., 2006) as implemented in Statistical Parametric Mapping toolbox (SPM12, Wellcome Trust Centre for Neuroimaging, London, UK) to model effective connectivity among distributed cortical sources within the sensorimotor network. The analysis was performed for two different periods 20–100 and 100–350 ms after the perturbation onset.

DCM estimates effective connectivity in a network of reconstructed cortical sources. DCM is a neurobiologically constrained source reconstruction scheme including both spatial forward modeling and model inversion (David et al., 2006). For the spatial forward model, DCM uses similar leadfields as other source reconstruction methods (Kiebel et al., 2006). Beyond other source reconstruction methods, DCM combines the spatial forward model with a biologically informed temporal forward model to estimate the connectivity between sources (Friston et al., 2003; David et al., 2006).

**Figure 1:**
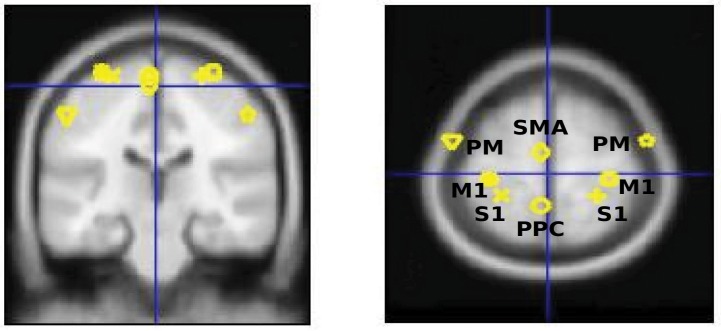
Eight selected cortical regions: left and right primary somatosensory cortex (S1), left and right primary motor cortex (M1), left and right bilateral premotor cortex (PM), and supplementary motor area (SMA), posterior parietal cortex (PPC).

**Figure 2:**
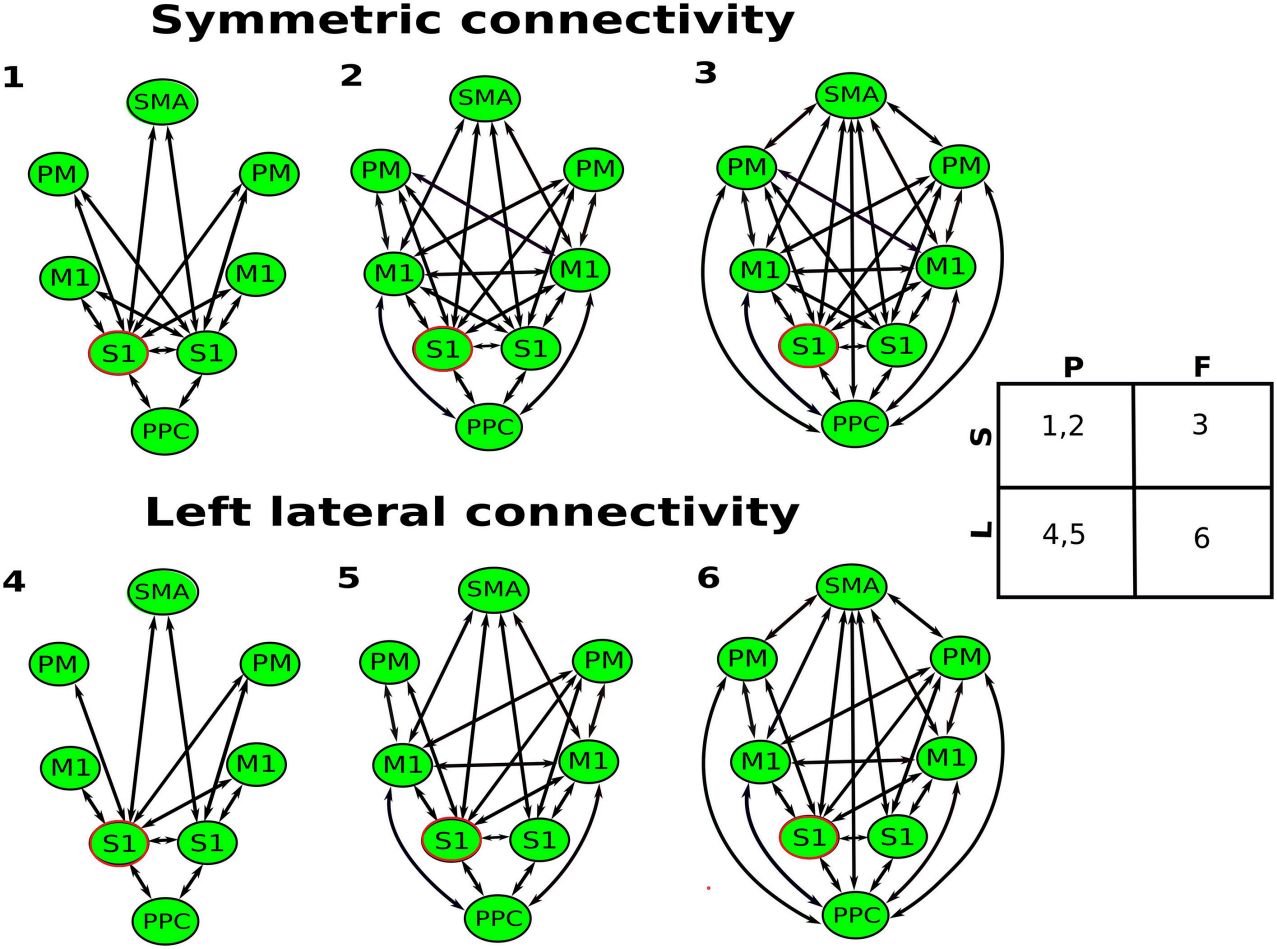
Six biologically plausible models for perturbation-modulated network. All of the models, the left S1 (marked in red) is the source receiving the external input (stretch of the flexor muscles of the right wrist). Model spaces are created using two attributes: partially (P) vs. fully (F) modulated by the stimulus, and left lateralized (L) vs. symmetric (S).

In this paper, the leadfield of each source is modeled by a single equivalent current dipole (Kiebel et al., 2006). DCM analysis requires users to specify the prior locations (in mm in MNI coordinates) of each source in the cortical network for building the spatial forward model (David et al., 2006). Based on the review from Scott (2002) (Scott, 2002), we selected eight key regions in the cortical sensorimotor network: left and right primary somatosensory cortex (S1), left and right primary motor cortex (M1), left and right bilateral premotor cortex (PM), supplementary motor area (SMA), and posterior parietal cortex (PPC). The MNI coordinates of hand/wrist regions in these eight cortical areas were informed by previous fMRI studies (Szameitat et al., 2012; Vlaar et al., 2016) and provided in Table 1. Based on these eight cortical sources (see Figure 1), we specified six different connectivity models as shown in Figure 2. In all of the models, the S1 is the source receiving the external input. The model space was created using two model attributes: (1) whether the connectivity is partially or fully modulated by the stimulus, and (2) whether interhemispheric connectivity is left lateralized (since the perturbation is given to the right wrist) or symmetric.

**Table 1:** MNI coordinates (mm) of eight sources in the cortical sensorimotor network: left (L) and right (R) primary somatosensory cortex (S1), left and right primary motor cortex (M1), left and right bilateral premotor cortex (PM), and supplementary motor area (SMA), posterior parietal cortex (PPC).

**Sources**	**MNI coordinates (mm)**
S1 L	−26 −40 68
S1 R	26 −40 68
M1 L	−33 −28 70
M1 R	33 −28 70
PM L	−54 −2 46
PM R	54 −2 46
SMA	−4 −10 64
PPC	−4 −46 68

The network model is inverted using a Bayesian approach described by Friston (2002), where a fix-form Laplace approximation is used to estimate probability distributions of parameters. This is under the Gaussian assumption, which enables computation of the likelihood from the prediction error. We then used Bayesian model comparison to identify the best model, based on approximation to the log-evidence obtained in the model inversion (Friston and Penny, 2011). In this study, we did not find an identical optimal model for all individuals. According to the practical recommendations provided by Stephan et al. (2010), the group-level analysis was performed to find the best cross-subject model, where the pooled log-evidence for each model (m_i_) across subjects (y_1_, …, y_7_) is defined as ln p(y_1_, …, y_7_|m_i_). Assuming that the data for different subjects are independent, we then have ln p(y_1_, …, y_7_|m_i_) = ln p(y_1_|m_i_) + ln p(y_2_|m_i_) +…+ p(y_7_|m_i_) (Penny et al., 2004). This pooled log-evidence indicates how well a particular model explains multiple datasets. To compare model evidences on group level, we used random-effect group Bayesian model selection (BMS). Classical random-effect analysis detects whether model evidence is consistent across subjects. In contrast, the group-BMS approach identifies the proportion of subjects, which is best described in terms of the model evidence, i.e., the posterior probability that each model is more frequent than others (Rigoux et al., 2014). The log group Bayes factor (ln BF_i, j_) between models is computed from pooled log-evidences, i.e., ln BF_i, j_ = ln (y_1_, …, y_7_|m_i_) -ln p(y_1_, …, y_7_|m_j_), to indicate that how much model *i* is superior to model *j* for the whole data set. The value of ln BF_i, j_ between 20 and 150 indicates a strong evidence (according to 95% confidence level) in favor of model i than model j, while ln BF_i, j_ larger than 150 indicates a very strong evidence (99% confidence level) (Penny et al., 2004).

After identifying the best cross-subject model (with the highest pooled log-evidence), we obtained the mean posterior estimates of all effective connectivity parameters for each subject and each period. These parameters represent the relative connectivity strengths between the two sources. The inferences on these parameters reflect the input (i.e., the muscle stretch) modulated changes in the effective connectivity. By investigating these inferences, we can identify the activities of which cortical areas are modulated by the muscle stretch and how they influence other cortical areas.

We averaged the connectivity strengths over subjects using Bayesian parameter averaging to get the mean estimate for each directional cortical interaction. We used one-sample *t*-test (two-tailed) to identify significant changes in the effective connectivity (*p* < 0.05, adjusted by false discovery rate estimation) in the best cross-subject model to get the perturbation-modulated effective connectivity for each period.

## Results

### Bayesian model selection

The effective connectivity in the cortical network after stretching the flexor muscles of the right wrist was modeled with DCM. We compared different Bayesian model families shown in Figure 2. Family-level Bayesian model comparison show that the left lateralized (L) models fit the data better than the symmetric (S) models for both periods (ln BF_L, S_ = 1,350 for 20–100 ms, and ln BF_L, S_ = 611 for 100–350 ms). The partial (P) modulated models fit the data better than the fully (F) modulated models for the period of 20–100 ms (ln BF_P, F_ = 1,706), while the fully modulated models provide substantially better fit for the period of 100–350 ms (ln BF_F, P_ = 3,670).

**Figure 3:**
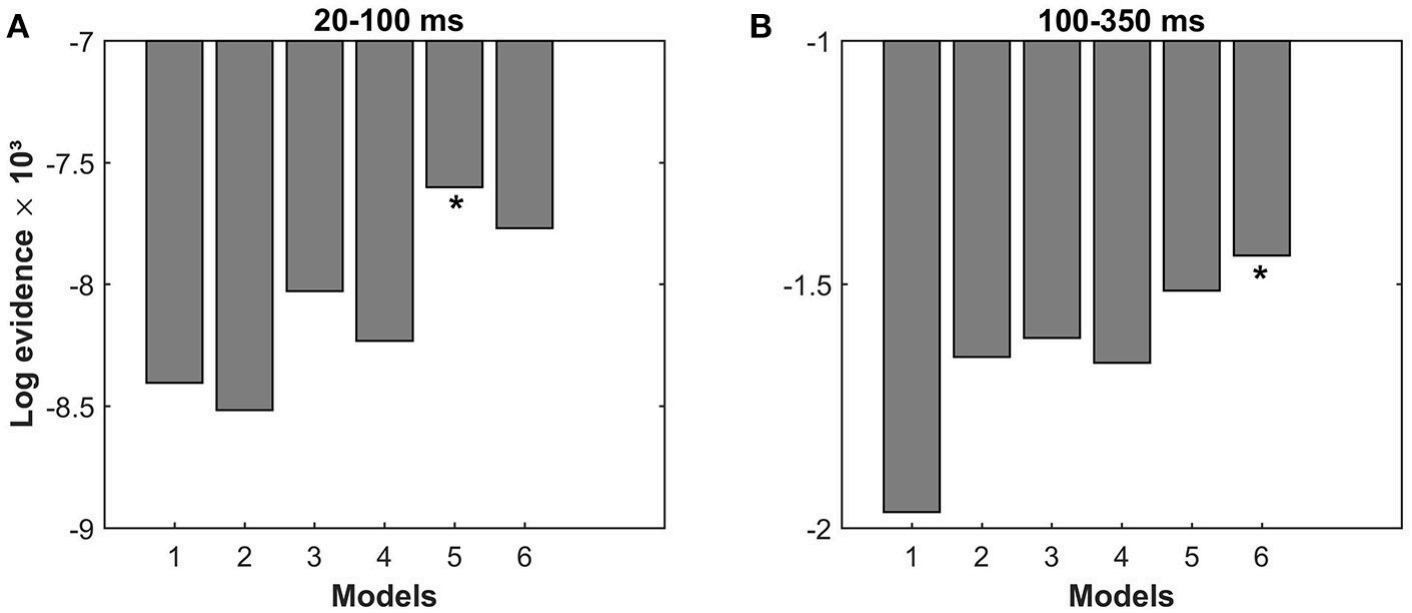
Results of Bayesian model selection. Comparison of the pooled log-evidences of the six models indicates: **(A)** Model 5 is the best model (log-evidence = −7,600) for the period of 20–100 ms and **(B)** Model 6 is the best model (log-evidence = −1,440) for the period of 100–350 ms.

Figure 3 shows the pooled log-evidences for different models. For the period of 20–100 ms, the model comparison shows the strongest evidence for Model 5, which is a partially modulated left lateral model, with a Bayes Factor (ln BF_5, 6_) of 168 over the second-best model (Model 6). For the period of 100–350 ms, the strongest evidence is present for Model 6, which is a fully modulated left lateral model, with a with a Bayes Factor (ln BF_6, 5_) of 72 over the second-best model (Model 5).

### Inference on coupling parameters

**Figure 4:**
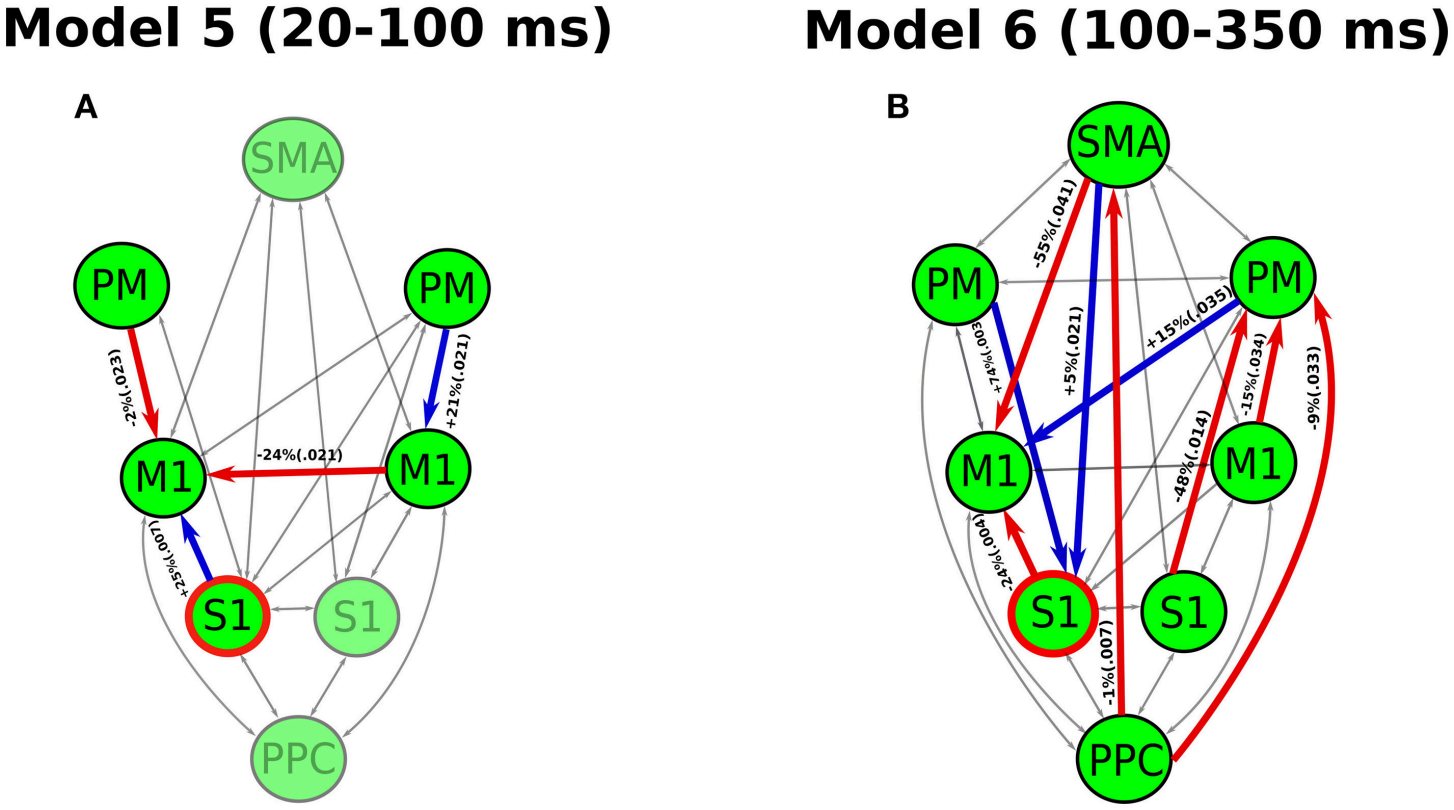
Modulatory effects of wrist muscle stretch on effective connectivity in the best model for **(A)** the period 20–100 ms post-perturbation and **(B)** the period of 100–350 ms post-perturbation. The significantly modulated effective connectivity and associated brain areas are highlighted in bold. The increased connectivity is indicated in blue, while the reduced connectivity is given in red. We also provide percentages of coupling change and *p*-value (in parentheses) for all the significantly modulated connectivity. The S1 (marked in red circle) is the source receiving the external input (stretch of right wrist).

The analysis of coupling parameters under the best cross-subject models reveals the significant modulations of effective connectivity by the perturbation for the period of 20–100 ms (Model 5) and 100–350 ms (Model 6), respectively (see Figure 4). During the period of 20–100 ms, the significant modulations only occur in the connectivity between M1 and a few cortical areas. In the left hemisphere (contralateral side to the perturbation), we detected a decrease in the effective connectivity from PM to M1 while an increase from S1 to M1. In the right hemisphere, only an increase of effective connectivity is shown from PM to M1. The cross-hemisphere interaction shows a reduced connectivity from right M1 to left M1. The left M1, which comprises the upper motoneurons of the right wrist muscles, appears a “sink” for all modulated connectivity pathways in this period.

During the period of 100–350 ms, a larger number of connections among more cortical areas is modulated by the perturbation. Different from the period of 20–100 ms, the connectivity with SMA, PPC and right S1 are also modulated. Specially, we found that a reduced connectivity pathway started from PPC through SMA to left M1. Additionally, there are three reduced connectivity pathways starting from PPC, left S1 and M1 all passing through right PM and arriving at left M1.

## Discussion

In this study, we investigated the response of the effective connectivity in the cortical network to stretch of the flexor muscles of the right wrist. We built model spaces with left lateralized (i.e., contralateral to the perturbed wrist) and symmetric models for comparison. We did not include right lateralized models, since all subjects are right-handed and the task is performed with the right wrist. DCM suggested strong evidence that contralateral (left) lateralized models were superior to the symmetric models for both rapid (20–100 ms) and slow (100–350 ms) periods. These results are in line with previous studies reporting contralateral hemisphere dominance of the cortical response to wrist perturbations (Campfens et al., 2015) and during motor control (Chen et al., 2010; Yang et al., 2016b).

### DCM for the early cortical response to muscle stretch

During the early period of 20–100 ms, the partially modulated models (of the effective connectivity in the cortical network) fit the data better than the fully modulated models, showing a relatively simpler network compared to the period of 100–350 ms. In the best model (Model 5), only a few connections among several key cortical areas are significantly modulated during the early period (see Figure 4A). This likely facilitates a rapid motor reaction to the perturbation without involving high level cognitive processes. Previous studies have found direct monosynaptic connections between the S1 and M1, which allows fast signal propagations between S1 and M1 (Rocco-Donovan et al., 2011). Here, we detected an increased connectivity from S1 to M1 in the contralateral hemisphere. This enhanced S1-M1 connectivity may lead to a quick sensory-motor processing in response to the unpredicted change (caused by the perturbation) in the sensory periphery.

A reduced connectivity from PM to M1 is shown at contralateral hemisphere in the early period. The PM is thought to be associated with predictions of sensory consequences of voluntary movements (Christensen et al., 2007). In the experiment, the subjects were required to maintain an isotonic wrist flexor torque before the perturbation. Thus, this voluntary control was accompanied with both the efferent motor command and an “efference copy” of this information (Wolpert and Flanagan, 2001). The communication between the M1 and PM is likely related to the cortical process of the efference copy to mediate movement predictions. This process may be inhibited due to the unpredicted change of sensory input, showing a decrease of effective connectivity from the PM to M1.

Additionally, a decreased effective connectivity is also shown from ipsilateral M1 to contralateral M1. The interhemispheric interaction of M1 has been reported by TMS and EEG studies during forearm muscle movement control (Ferbert et al., 1992; Bönstrup et al., 2016). This interhemispheric inhibition is thought to be related to the activity of inhibitory GABA-ergic interneurons (Daskalakis et al., 2002) to prevent the interference from the opposite hemisphere (e.g., mirror movement) during movement control (Mayston et al., 1999). Thus, this inhibitory effect may facilitate the cortical response to the unpredictable perturbation without the interruption of ipsilateral M1. All the information of modulation eventually flows into the contralateral M1 which allows the early cortical activity to be transmitted to the motor units through the monosynaptic corticospinal connection (Nielsen, 2016). This is the fastest cortical pathway contributing to the muscle stretch response, which likely lead to the rapid transcortical muscle response.

### Effective connectivity during the late cortical response to muscle stretch

In the late period of 100–350 ms, there are more cortical areas and connections are modulated (see Figure 4B). In particular, the connection between PPC and SMA is modulated, indicating that these cortical areas may play important roles in the late cortical responses to muscle stretch. The PPC is thought be involved in the multisensory integration and coordinate transformations from sensory inputs to motor outputs during feedback-based movement control (Andersen and Buneo, 2002). The SMA is crucial for linking cognition to motor action (Nachev et al., 2008). The modulation of PPC-SMA connectivity indicates a high-level cognitive process for the slow, voluntary response, which is not shown for the early period. The reduced connectivity from PPC to SMA likely indicates a negative feedback in sensorimotor control loop. This negative feedback may play a role in correcting the motor actions based on the integrated sensory information. Besides, multiple pathways ending at the contralateral M1 are modulated in this period, indicating rich communications between different cortical areas. The complexity of this network in late period likely delays the voluntary motor output to facilitate the coordinated (slow) muscle responses.

## Conclusion

Muscle stretch modulates different effective cortico-cortical connections during early (before 100 ms post-perturbation) and late (after 100 ms) periods of cortical responses. Only a few effective cortico-cortical connections are modulated in the early period, while more cortical areas are involved in the late period with more effective connections modulated. The limited early cortical network likely allows for a rapid muscle response without involving high-level cognitive processes. The complexity of the late network may delay the voluntary motor output from the cortex, so as to facilitate the coordinated responses in the “standard” voluntary reaction to muscle stretch.

## Author contributions

YY and AS contributed in problem identification. BG conducted the data analysis under the supervision of YY and AS. YY and BG drafted the manuscript. YY and AS edited the manuscript.

### Conflict of interest statement

The authors declare that the research was conducted in the absence of any commercial or financial relationships that could be construed as a potential conflict of interest.
